# Drug-induced activation of “junk” DNA – A path to combat cancer therapy resistance?

**DOI:** 10.18632/oncoscience.364

**Published:** 2017-10-01

**Authors:** Marie Classon, Kelly LaMarco, Daniel D. De Carvalho

**Affiliations:** Princess Margaret Cancer Centre, University Health Network, Toronto, Canada; Department of Medical Biophysics, University of Toronto, Toronto, Canada

**Keywords:** epigenetic therapy, repetitive elements, cancer therapy resistance

There is currently a vast array of anti-cancer therapies, including traditional cytotoxic drugs, targeted kinase inhibitors and a growing number of immunotherapies, that function by ‘reinvigorating’ a patient's own immune system to attack tumor cells. Despite this broad range of “weapons”, innate and acquired resistance to therapy remains a major impediment to long term remission or cure. Several recent reports have put forward a model in which differential activation of genomic transposable elements (TEs), such as endogenous retroviruses (ERVs) and long interspersed elements (LINE-1), by epigenetic drugs can accentuate cancer treatments.

Activation of TEs, which account for a large fraction of the human genome, can result in genomic alterations (mutations and chromosomal re-arrangements) and changes in gene transcription. Because excessive TE activation can incite genomic instability or create auto-immunity, these DNA regions are normally subject to stringent control by DNA methylation and other repressive mechanisms in most somatic tissues. However, it has long been appreciated that tumor cells as well as aging tissues display DNA hypomethylation in genomic regions that contain TEs. Considering that cancer is, in part, an aging-related disease, it is noteworthy that inappropriate activation of TEs has been suggested to be involved in cancer development.

Recent studies have shown that epigenetic therapies, including DNA hypomethylating agents and histone deacetylase inhibitors (HDACi), can increase the expression of TEs in treated cancer cells [1-3, 6]. Furthermore, these studies show that increased TE expression negatively affects cancer cell survival due to the formation of dsRNA and downstream activation of the cytosolic RNA sensing pathway and an IFN (interferon) signature, mimicking a viral infection [1,2]. “Viral mimicry” and/or presentation of newly expressed tumor antigens induced by TE activation could also play a role in immune-mediated cancer cell clearance, as suggested by studies in mouse models in which DNA hypomethylating agents increase the response to immunotherapy [2]. Based on these findings, several combination immunotherapy clinical trials have been initiated using such epigenetic therapies.

Studies in colorectal tumor cells also demonstrated that DNA hypomethylating agents negatively affect their tumor re-initiating potential [1] - a phenotype associated with the “cancer stem cell” paradigm [4]. In this context, we note that leukemic stem cells, which demonstrate increased resistance to therapy and might serve as reservoirs of relapse, exhibit significant transcriptional repression of TEs and IFN–induced pathways as compared to other leukemic cells [5]. Consistent with these observations, a recent study found that DNA hypomethylating agents and HDAC inhibitors can negatively affect the survival and/or propagation of a small subpopulation of cancer cells (with cancer stem cell features) that persist during otherwise lethal drug exposures [6]. These drug-tolerant persister cells (DTPs) exhibit epigenetically mediated repression of some TEs, which are induced by cancer drugs in the heterogeneous population [6]. The decreased number of DTPs seen following epigenetic therapy is seemingly due to a combination of increased genome instability (most likely driven by activation of TEs including LINE-1), as well as the activation of the viral defense machinery [6].

This study also shows that the repression of drug-induced LINE-1 expression in DTPs is in part imposed by H3K9me3-mediated heterochromatin formation [6], a mechanism previously shown to be involved in maintaining genome stability in developmental settings where DNA methylation is decreased. For example, the H3K9-methyltransferase SETDB1 serves to promote genomic integrity in primordial germ cells [7]. Interestingly, Acute Myeloid Leukemia (AML) cells may utilize a similar SETDB1-dependent mechanism for their survival, and tumor cells generally express increased levels of SETDB1 as compared to normal tissue [8]. These studies suggest that, in addition to DNA hypomethylating agents and HDAC inhibitors, tumor-specific TE repression mechanisms, not used in adult somatic cells (and perhaps “borrowed” from developmental biology), could be harnessed for future drug development aimed at potentiating therapies.

Collectively, these studies point to a novel paradigm in which dysregulated expression of TEs affects a variety of cancer therapy responses including those to targeted agents and immunotherapies. In the case of immunotherapy responses, future studies are likely to determine whether tumor specific activation of TEs or use of drugs that interfere with the viral response machinery will be more effective as treatment combinations. It is also noteworthy that any contemplated therapeutic regimen that might render tumor cells more visible to the immune system should not have detrimental effects on relevant immune cell functions, nor should it affect normal tissues. It is also important to consider that activation of TEs and other repeat elements can provide the cancer cell with endless abilities to adapt to new environments by altering their genomes – a phenomenon first described in maize kernels by Barbara McClintock more than 70 years ago.

**Figure 1:**
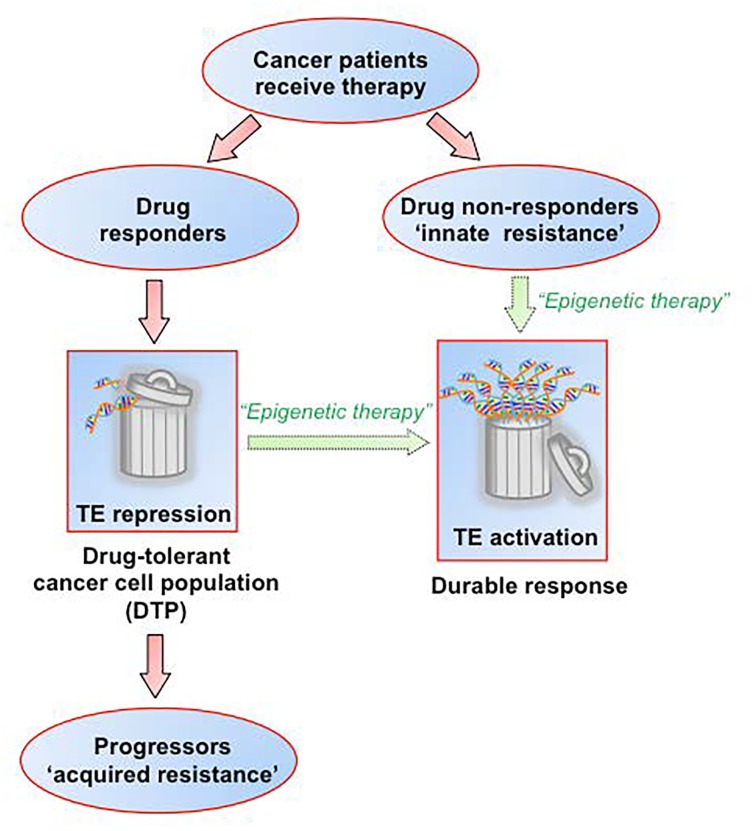
Can epigenetic modulation of the expression of genomic repetitive elements (long considered “junk” DNA) boost therapeutic response in cancer patients?
